# Autonomous control of cardiovascular reactivity in patients with episodic and chronic forms of migraine

**DOI:** 10.1186/s10194-016-0645-6

**Published:** 2016-05-11

**Authors:** Oleg V. Mamontov, Laura Babayan, Alexander V. Amelin, Rashid Giniatullin, Alexei A. Kamshilin

**Affiliations:** Almazov Federal Heart, Blood and Endocrinology Centre, St. Petersburg, Russia; Pavlov First Saint Petersburg State Medical University, St. Petersburg, Russia; Department of Neurobiology, University of Eastern Finland, Kuopio, Finland; Kazan Federal University, Kazan, Russia; Department of Computer Photonics and Videomatics, ITMO University, St. Petersburg, Russia

## Abstract

**Background:**

The autonomous cardiovascular control can contribute to progression of migraine. However, current data on cardiovascular reactivity in migraine, especially severe forms, are essentially contradictory. The main aim of this study was to compare the autonomous regulation of circulation in patients with episodic and chronic migraine and healthy subjects.

**Methods:**

Seventy three migraine patients (mean age 35 ± 10) including episodic migraine (51 patients, 4–14 headache days/months) and chronic migraine (22 patients, ≥15 headache days/month) along with age-match control (71 healthy voluntaries) were examined. The autonomic regulation of circulation was examined with the tilt-table test, a deep breathing and Valsalva Maneuver, handgrip test, cold-stress vasoconstriction, arterial baroreflex and blood pressure variability.

**Results:**

The changes in heart rate induced by deep breathing, Valsalva Maneuver, and blood pressure in tilt-table test in patients with migraine did not differ from the control group. In contrast, the values of cold-stress-vasoconstriction forearm blood-flow reactivity (*p* <0.001), the increase in diastolic blood pressure in handgrip test (*p* <0.001), mean blood pressure in the late stage of the second phase of Valsalva Maneuver (*p* <0.001) and blood pressure variability (*p* <0.005) were all higher in patients with migraine than in the control group.

**Conclusion:**

Thus, both episodic and chronic migraine are associated with significant disturbances in autonomous control resulting in enhanced vascular reactivity whereas the cardiac regulation remains largely unchanged.

## Background

The activation of the trigeminovascular system comprising somatic and autonomous nerves and meningeal vessels, plays a central role in the pathogenesis of migraine [1–3]. During migraine attack initial vasodilatation is often following by the constriction of densely innervated vessels in dura mater [4], an origin site for headache-related nociceptive firing [5–7]. However, pathophysiological mechanisms leading to migraine chronization remain largely unknown. It is also remains unclear whether the chronic migraine is associated with different neuronal mechanisms including function of autonomous nerves [8]. Current evidence suggests that pathophysiologic mechanisms of migraine persist even in the interictal period [9, 10]. Furthermore, it has been suggested that migraine is a manifestation of a systemic vasculopathy [11]. Consistent with this view, much attention was paid previously to changes in the vascular system controlled by autonomic nervous system [12–14], which plays an important role in the regulation of the heart rate and vascular tone. The abnormal autonomous control as the contributing factor for migraine was discussed in many previous studies [9, 13, 15–17]. In particular, it has been suggested that the lack of vasoconstriction may predispose to migraine pain [15]. The evidence suggests that, even in the interictal period, migraineurs have reduced catecholamine levels in the rest and during activation of the sympathetic nerves [18, 19]. However, other data suggest that the reactivity of vessels in response to alpha-1 agonist phenylephrine is increased consistent with the enhanced blood pressure response during the orthostatic load [20].

Conclusions on the reduced sympathetic reactivity are often based on results of the orthostatic load and handgrip test, which, however, are not mediated by the sympathetic nerves exclusively as evidenced by results of microneurography [21]. Cold stress is also used to evaluate activity of the sympathetic nerves [22, 23]. We have previously shown that patients with a predisposition to headaches have a reduced temperature of the nose (“cold nose” [16]) and the extremities [17]. Increased sympathetic vascular tone should increase the peripheral vascular resistance, which can be evaluated by occlusion plethysmography of cutaneous vessels in the forearm [24, 25]. Thus, the assessment of blood flow in response to cold stress is the appropriate method to study the vasomotor reactivity in clinical settings. In addition, vasomotor reactivity may be evaluated by the variability of the blood pressure based on spectral analysis [26]. Thus, to assess the sympathetic control, in the present study, we evaluated the vasomotor reactivity of forearm vessels to cold stress along with variability of the blood pressure in patients at least 48 hours after the last attack.

In contrast to variable results with sympathetic control, many studies indicated reduced parasympathetic control in migraine [27–29]. In particular, cardiovascular reflexes evaluated with the Valsalva Maneuver [27] or with heart rate variability [16, 29] suggested a parasympathetic hypofunction in migraine. However, one recent study revealed an enhanced level of the parasympathetic co-transmitter vasointestinal peptide as a possible biomarker of migraine chronization [30]. Therefore, our aim was to study the state of the autonomous control in patients with episodic and chronic migraine using a battery of tests directed to evaluate the cardiac and vascular reactivity.

## Methods

### Study groups

The study group involved 73 patients (10 males and 63 females), aged 35.3 ± 10.1 years, and consisted of patients with frequent episodic (*n* = 51) and chronic migraine (*n* = 22). All migraine patients involved in this study were primary patients who consulted our medical center for the first time. They did not receive preventive treatment at least two months before the study. The treatment was prescribed after the study to exclude its influence on the research results. Only five patients of the group of episodic migraine and one of the group of chronic migraine were suffering from the migraine with aura. Chronic migraine patients had no triptan overuse. The diagnosis of migraine was made using the criteria of the International Classification of Headache Disorders, 3rd edition (beta version) [31]. Patients with hypertension were excluded from this study since previous studies indicated a complex link between hypertension and migraine [32].

Patients had no clinically significant comorbidities. They did not take medicines affecting circulation or interfering with autonomous control in the day of investigation, and the day before. The control group included 71 age-matched (mean age 35.3 ± 12.1 years) healthy volunteers (14 males and 74 females). All patients and volunteers were between 18 and 50 years old. The basic clinical characteristics of both groups are presented in the Table 1. Whereas the basic anthropometric characteristics of these groups did not differ, the systolic blood pressure in patients with migraine was lower than in the control group without differences in diastolic blood pressure and heart rate (Table 1). All patients underwent a combined investigation of the autonomous regulation of circulation, including:the tilt-table test (TT), a deep-breathing (DB) and Valsalva Maneuver (VM), handgrip test (HG), cold-stress vasoconstriction (CSV), arterial baroreflex (BRS) and blood pressure variability (BPV). All the studies, except the tilt test were performed in the supine position. Recovery time between successive tests was at least 5 min to ensure both the heart rate and the vascular tone recovery after an exercise [33, 34]. Assessment of the circulation was carried out with the non-invasive beat-to-beat blood pressure monitor Finometer-Pro (FMS, Holland) along with the parallel recording of the ECG. The forearm blood flow was measured by venous occlusion plethysmography using Dohn air-filled plethysmograph.

**Table 1:** General description of patients

Parameter	Episodic migraine	Chronic migraine	Control group
	*n* = 51	*n* = 22	*n* = 71
Age, years	35.2 ± 9.7	36.2 ± 10.8	35.3 ± 11.9
Gender m/f	8/43	3/19	14/74
Body mass index, kg/m^2^	22.0 ± 3.9	22.1 ± 4.4	22.1 ± 3.3
Heart rate, bpm	76.4 ± 9.6*	70.0 ± 8.7*	72.9 ± 11.9
Systolic blood pressure, mmHg	117.0 ± 9.9	117.5 ± 9.6	120.1 ± 9.2
Diastolic blood pressure, mmHg	68.8 ± 6.9	69.7 ± 7.7	66.9 ± 9.6
Migraine with aura and without aura	5/46	1/21	-
Use of analgesics, %	94 %	95 %	No

Legend: * significant difference (*p* < 0.017) between two migraine groups with correction Bonferroni

### Protocol of the study

Research plan included the following sequence of tests:

1. *Tilt-test* is capable to evaluate the activity of the sympathetic nervous system directed to maintain the hemodynamic parameters despite a decrease in diastolic refilling due to the reduction of venous return [35, 36]. The tilt-test was performed in the short version comprising 10 min of rest following by the orthostatic load during next 10 mins. Orthostatic load was performed on a tilt table, inclined at the angle of 70° as it was described earlier [36]. The blood pressure and the heart rate were measured for the entire period of the orthostatic load.
2. *The deep breathing test* allows estimation of mainly the parasympathetic regulation of the heart mediated by the vagus nerve [36]. It should be noted, however, that the sympathetic nerves can also affect the breathing rate [37]. The deep breathing test took 1 min at the rate of 6 slow breaths/min performed in the supine position. Expiration/inspiration coefficient was calculated from the ratio of longest and shortest RR intervals during 6 respiratory cycles.
3. The standard *Valsalva Maneuver* designed to study the autonomic regulation of heart rate, as well as neurogenic vascular reactivity [36]. To perform this test, the patient produced the forced exhalation through the mouthpiece connected to the manometer during 15 s against the resistance of 40 mmHg as suggested earlier [36]. The heart rate and the mean blood pressure were recorded continuously before, during, and 30 s after the expiration. Valsalva index (reporting cardiac function) was calculated as the ratio of the longest to the shortest RR interval during the VM. Also the time course of the mean blood pressure (reporting vascular reactivity) was assessed at the late stage of the second phase of this Maneuver. The blood pressure increase at the end of the second phase of VM indicates the sympathetic reactivity in response to deactivation of the arterial baroreflex, which occurs due to a decrease in cardiac output [38].
4. *Handgrip test* measures changes in the level of diastolic blood pressure, which reflects the neurogenic vascular reactivity providing increased vascular resistance in response to mechanical stimulation of the muscle afferents [39]. This test was performed by applying 30 % of the maximum force on the handle dynamometer for 3 min to measure the diastolic blood pressure, which is a modification of the technique described by Ewing [39]. The diastolic pressure level in the last 30 s of rest was compared with results of the loading period.
5. *Сold-stress induced vasoconstriction* reporting sympathetic cardiovascular responses independently from baroreflex function [40] was carried out by the application of a cold ice pack to the chest area for two mins to evaluate changes in the blood flow in the forearm with occlusion plethysmography. Plethysmographic recording were performed every 8 s to estimate blood flow at the baseline and during cold stress induction. Blood flow parameters were calculated as the average value from 2–4 individual measurements. The relative decline in parameters during cold stress was compared with the baseline values.
6. Both the spontaneous arterial baroreflex, which reflects parasympathetic cardiovascular regulation [41], and beat-to-beat blood pressure variability reflecting mainly the neurogenic control of the vascular tone [42] were estimated in patients at the rest in supine position.

### Statistical analysis

Statistical analysis was performed using the software STATISTICA 10. The comparative analysis was carried out by using the parametric *T*-test for independent sample. To assess the relationship between indicators of vasomotor regulation, Pearson correlation coefficients were used. All data are presented as the mean value ± standard deviation (SD). Bonferroni correction was applied for comparative analysis of three groups with the significance level *p* < 0.017. Nevertheless, for comparison between two migraine groups and the control the difference was assumed to be significant when *p* < 0.025.

## Results

The general characteristics of the patients in both groups and the control group are shown in Table 1. As it follows from the Table 1, no differences were found between both patient groups and the control group, except the lower level (*p* < 0.017) of the heart rate in patients with chronic migraine compared to the episodic migraine.

### Cardiac regulation

The cardiac regulation in episodic and chronic migraine was addressed by performing the deep breathing test, calculating the Valsalva index, and by measuring the arterial baroreflex. However, we found that the heart rate changes induced by the deep breathing, the Valsalva index, or arterial baroreflex, as well as the initial heart rate, were not different between both migraine groups and control (*p* > 0.017 for all parameters, Table 2). These data indicated that the cardiac regulation remained remarkably stable in frequent episodic or chronic migraine despite the severity of this disorder.

**Table 2:** Parameters of autonomic regulation in patients with migraine and in control group

Tests concerning the cardiac control		Episodic migraine	Chronic migraine	Control group	*p*
		*n* = 51	*n* = 22	*n* = 71	
	Arterial baroreflex, ms/mmHg	11.5 ± 6.8	14.4 ± 10.0	13.1 ± 8.1	*p >* 0.017
	Valsalva index, arbitrary units	2.2 ± 0.4	2.1 ± 0.4	2.1 ± 0.5	*p >* 0.017
	Expiration/inspiration ratio in deep breathing test	1.36 ± 0.15	1.36 ± 0.19	1.35 ± 0.20	*p >* 0.017

### Vasomotor regulation

**Fig. 1:**
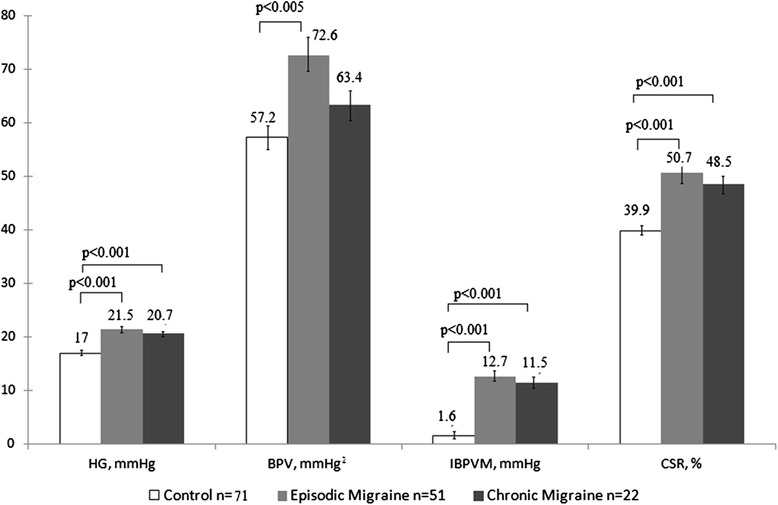
Parameters of neurogenic vascular reactivity. The numbers show mean values while the error bars represent the standard error of the mean. Legend: HG – handgrip test, CSV – cold-stress vasoconstriction, IBPVM – increment of mean blood pressure during Valsalva Maneuver, BPV – blood pressure variability

Next, we measured the vasomotor reactivity by applying the orthostatic load tilt-table test, Valsalva Maneuver (2^nd^ stage), handgrip test, cold-stress vasoconstriction, and measured the blood pressure variability. Unlike the cardiac regulation, in vascular reactivity patients in both migraine groups (episodic and chronic migraine) were different from the control group. Thus, in four of these tests, we obtained significant differences between the control and migraine groups. The strongest effect (*p* < 0.001) was observed in the later stage of the second phase of the Valsalva Maneuver when the mean blood pressure had 3-fold increase in episodic and chronic migraine groups compared to the control (Fig. 1). Likewise, in the handgrip test, the growth of the diastolic blood pressure in migraineurs from both groups was also essentially more pronounced (*p* < 0.001). Cold-stress vasoconstriction demonstrated highly significant (*p* < 0.001) changes in the episodic and chronic migraine groups, as well (Fig. 1). All the denoted parameters differ significantly from the control group considering Bonferroni correction (*p* < 0.017. We also found the increased blood pressure variability in the episodic migraine group (*p* = 0.023), which shows vascular reactivity mediated by sympathetic efferent nerves. However, in the group of chronic migraine this parameter was not significantly different from the control group (Fig. 1).

**Fig. 2:**
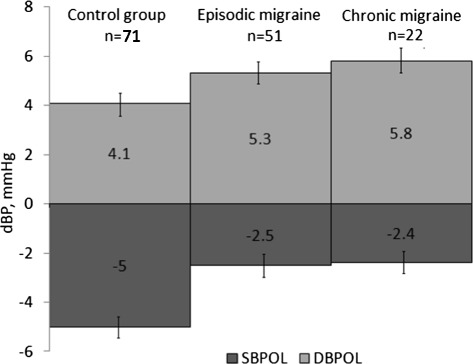
Changes in the systolic and diastolic blood pressure during tilt table test in migraine and control groups. The numbers show mean values while the error bars represent the standard error of the mean. Legend: SBPOL – systolic blood pressure orthostatic load, DBPOL – diastolic blood pressure orthostatic load. There is non-significant difference (*p* > 0.05) between each of migraine groups and the control group

The tilt table test was the only one, in which no differences was revealed between the both migraine groups and the control group for both systolic and diastolic pressure changes (*p* > 0.025 in all cases, Fig. 2).

### Correlations between parameters of vascular reactivity

In order to explore the consistency between results obtained from different tests we carried out a correlation analysis of different parameters of vascular reactivity. As we obtained similar changes in parameters inside of both migraine groups in order to increase the power of this analysis, we pooled these data in one ‘migraine group’. Our correlation analysis revealed that in the control group the results of the handgrip test, the increment of mean blood pressure in the second stage of Valsalva Maneuver, and the cold-stress vasoconstriction strongly correlated with the blood pressure variability (*p* < 0.01, Table 3). Similar strong correlation was also found in the migraine group indicating a persistence of these enhanced functions in disease.

**Table 3:** Pearson correlation coefficients of vasomotor regulation in the migraine group (upper part) and the control group (lower part)

Migraine group						
	HG	CSV	SBPOL	DBPOL	IBPVM	BPV
HG	1	0.17	0.09	0.04	0.17	**0.35**
		*p* = 0.21	*p* = 0.48	*p* = 0.7	*p* = 0.20	***p*** **< 0.01**
CSV	0.17	1	**0.29**	0.22	0.13	**0.38**
	*p* = 0.21		***p*** **< 0.05**	*p* = 0.089	*p* = 0.322	***p*** **< 0.005**
SBPOL	0.09	**0.29**	1	**0.81**	−0.19	−0.01
	*p* =0.48	***p*** **<0.05**		***p*** **< 0.001**	*p* = 0.143	*p* <0.91
DBPOL	0.04	0.22	**0.81**	1	−0.25	0.02
	*p* = 0.74	*p* = 0.09	***p*** **< 0.001**		*p* = 0.05	*p* = 0.87
IBPVM	0.17	0.13	−0.19	−0.25	1	**0.42**
	*p* = 0.2	*p* = 0.3	*p* = 0.1	*p* = 0.05		***p*** **< 0.001**
BPV	**0.35**	**0.38**	−0.01	0.02	**0.42**	1
	***p*** **< 0.01**	***p*** **< 0.005**	*p* = 0.91	*p* = 0.87	***p*** **< 0.001**	
Control group						
	HG	CSV	SBPOL	DBPOL	IBPVM	BPV
HG	1	0.14	0.13	0.09	0.19	**0.33**
		*p* = 0.27	*p* = 0.30	*p* = 0.50	*p* = 0.13	***p*** **< 0.01**
CSV	0.14	1	**0.25**	0.16	0.06	**0.42**
	*p* = 0.27		***p*** **< 0.05**	*p* = 0.23	*p* = 0.66	***p*** **< 0.001**
SBPOL	0.13	**0.25**	1	**0.82**	−0.10	−0.02
	*p* = 0.30	***p*** **< 0.05**		***p <*** **0.001**	*p* = 0.415	*p* = 0.903
DBPOL	0.09	0.16	**0.82**	1	−0.13	−0.01
	*p* = 0.50	*p* = 0.23	***p*** **< 0.001**		*p* = 0.31	*p* = 0.94
IBPVM	0.19	0.06	−0.10	−0.13	1	**0.35**
	*p* = 0.13	*p* = 0.66	*p* = 0.42	*p* = 0.31		***p*** **< 0.005**
BPV	**0.33**	**0.42**	−0.02	−0.01	**0.35**	1
	***p*** **< 0.01**	***p*** **< 0.001**	*p* = 0.903	*p* = 0.942	***p*** **< 0.005**	

Legend: *HG* handgrip test, *CSV* cold-stress vasoconstriction, *SBPOL* systolic blood pressure orthostatic load, *DBPOL* diastolic blood pressure orthostatic load, *IBPVM* increment of mean blood pressure during Valsalva Maneuver, *BPV* blood pressure variability, significant correlation is highlighted by bold fonts

## Discussion

In this study, using multiple testing in the interictal period we found significant differences in autonomic regulation of circulation in patients with episodic and chronic migraine comparing with control group. The identified differences concerned primarily the vasomotor reactivity, which was largely enhanced, while no changes in cardiac regulation were found in the migraine group. Interestingly, in chronic migraine these disturbances were not further aggravated comparing with episodic migraine. These vasomotor disturbances could contribute to the persistence of episodic and chronic migraine and suggest new potential targets for the complex therapy of this disorder via correction of autonomous regulation.

### Dysfunctional vasomotor reactivity in episodic and chronic migraine

The pathophysiological mechanisms leading to migraine chronization remain largely unknown. One popular view suggests that chronic migraine evolves from episodic migraine in susceptible individuals [8] with the rate of about 3 % per year [43]. However, recent data suggest that chronic migraine, which is a separate disorder according to ICHD-3beta, in contrast to episodic migraine, is based on different neurological mechanisms [44]. One of most recent studies, performed in a big Italian sample (3500 participants) revealed that the chronic migraine (≥15 days/month) was reported by 7.0 % of participants [45]. Interestingly, that there was a large (more than in migraine in general) prevalence of females with chronic migraine (10.6 % versus 2 % of males) and this was often associated with medication overuse migraine. Our current study was focused on episodic and chronic migraine mainly in females (63 of 73 patients) and, in the interictal period, we found essentially modified autonomic regulation in almost all patients, primarily in vascular control consistent with view that migraine is associated with the systemic vasculopathy [11].

The studies on the role of the autonomic nerves system in primary headaches have a long history and some autonomic disturbances are even presented as one of criteria distinguishing migraine from other types of headache [31]. However, few of these studies were devoted to these changes in chronic migraine. One recent study reported that migraineurs have higher parasympathetic influence on the heart rate compared to the control group [30]. With several tests used in our study, we found no significant changes in parameters characterizing the autonomic regulation of the cardiac function.

However, we found in almost all tests, reflecting the vascular reactivity that there were significant differences in neurogenic vascular regulation in migrainers of both groups compared to control. Thus, one of the most significant changes was found in the second stage of the Valsalva Maneuver. The latter, likely reflects the enhanced sympathetic neuronal activity directed to compensate a significant decrease in the venous return and to keep the normal level of the blood pressure, which requires the rapid increase of the vascular tone [46]. Thus, this enhanced response in the migraine group likely reflects the augmented role of the sympathetic nerves.

Consistent with activation of the sympathetic nerves, we also observed, in patients with migraine, the enhanced cold induced vasoconstriction [47]. The cold stress, based on the simple reflex, reflects the general neuro-vascular reactivity rather than the contribution of the other components of the cold stress [48]. Interestingly, no changes of systolic or diastolic blood pressure were found in migraineurs treated with the tilt test. The relative insensitivity of the orthostatic load in migraine can be explained by the limited specificity of this test for evaluation of the vascular reactivity because compensation of the orthostatic load is mediated by various mechanisms, and most of them do not directly require activation of the sympathetic nervous system.

An increase of blood pressure in the second stage of the Valsalva Maneuver is a sign of the enhanced baroreflex vascular reactivity. Similarly, increased vasoconstrictive reaction in the cold test is a direct consequence of the raised vascular resistance, which is mediated by the efferent sympathetic nerves. Notably, all parameters of vascular reactivity both in control and in both migraine groups positively correlated with blood pressure variability. This likely reflects the fact, that each of these parameters is the essential contributor to the variability of the blood pressure in health and disease.

### Sympathetic regulation versus parasympathetic

Since early studies of the autonomic regulation in migraine, there are still conflicting data on the role of sympathetic versus parasympathetic nerves in this disorder. In particular, cardiovascular reflexes evaluated with the Valsalva Maneuver [27] or with heart rate variability [16, 29] suggested a parasympathetic hypofunction in migraine whereas others found no changes. Some of these contradictions were overviewed by Yerdelen et al. [49] who did not find changes in the parasympathetic control in episodic migraine like previous study by Mosek et al. [22] The novelty of our study, in addition to the wide range of tests used here, was that we focused on the little understood episodic and chronic migraine. In these study groups, based on the data of cardiac tests, we found, similar to above-mentioned studies, that the parasympathetic control was almost unchanged.

In summary, our findings of similar dysfunctional autonomic vascular control in the chronic and episodic migraine groups are consistent with the view that likely other factors including comorbidities with psychiatric and gastrointestinal diseases are determining the profile of patients suffering (or predisposed) to chronic migraine [8, 50]. Therefore, based on similar presentation of disrupted autonomous control we can suggest that these disturbances are reflecting the very early autonomous abnormalities related to migraine. However, since this study was performed in pain free period, we cannot exclude that even more profound differences between the migraine and control groups and between chronic and episodic migraine could develop during attacks.

### Limitations of the study

Although the data presented here are not robust, all our patients underwent the multiple testing, which is essentially time consuming. Despite the numerous tests used in this study, they reflect the vasomotor reactivity only indirectly. The direct approach for the study of the sympathetic response in migraine would be the recording of the activity of the sympathetic nerves, which however has a limited use in the clinical practice due to the invasive nature of this procedure. In addition, vascular regulation may change during acute attacks, which would represent, however, the aim and the target of the future separate studies.

## Conclusion

In conclusion, our study revealed that in patients with episodic or chronic migraine without hypertension the cardiac reactivity almost did not differ from the control group, whereas the vascular reactivity was largely enhanced. The most significant differences in the vascular reactivity in patients with migraine were presented as the more significant growth of the blood pressure at the later stage of the second phase of the Valsalva Maneuver and in the response of the blood flow to the cold stress. Unchanged orthostatic blood pressure response in patients with migraine may be associated with the limited specificity of this test.

### Ethics statement

This study was conducted in accordance with ethical standards presented in the 1964 Declaration of Helsinki. The protocol of this study was approved by the Ethics Committee of the Pavlov First Saint-Petersburg State Medical University. All subjects provided a written consent for this study.

## Abbreviations

BPV: blood pressure variability
BRS: arterial baroreflex
CSV: cold-stress vasoconstriction
DB: deep breathing
DBPOL: diastolic blood pressure orthostatic load
HG: handgrip test
IBPVM: increment of mean blood pressure during Valsalva Maneuver
SBPOL: systolic blood pressure orthostatic load
TT: tilt-table test
VM: Valsalva Maneuver
